# Enhancing gene regulatory network inference through data integration with markov random fields

**DOI:** 10.1038/srep41174

**Published:** 2017-02-01

**Authors:** Michael Banf, Seung Y. Rhee

**Affiliations:** 1 Department of Plant Biology, Carnegie Institution for Science, 93405 Stanford, USA

## Abstract

A gene regulatory network links transcription factors to their target genes and represents a map of transcriptional regulation. Much progress has been made in deciphering gene regulatory networks computationally. However, gene regulatory network inference for most eukaryotic organisms remain challenging. To improve the accuracy of gene regulatory network inference and facilitate candidate selection for experimentation, we developed an algorithm called GRACE (Gene Regulatory network inference ACcuracy Enhancement). GRACE exploits biological *a priori* and heterogeneous data integration to generate high- confidence network predictions for eukaryotic organisms using Markov Random Fields in a semi-supervised fashion. GRACE uses a novel optimization scheme to integrate regulatory evidence and biological relevance. It is particularly suited for model learning with sparse regulatory gold standard data. We show GRACE’s potential to produce high confidence regulatory networks compared to state of the art approaches using *Drosophila melanogaster* and *Arabidopsis thaliana* data. In an *A. thaliana* developmental gene regulatory network, GRACE recovers cell cycle related regulatory mechanisms and further hypothesizes several novel regulatory links, including a putative control mechanism of vascular structure formation due to modifications in cell proliferation.

Transcriptional regulation is essential to life and is orchestrated by complex arrays of protein and RNA molecules. The most basic type of transcriptional regulation is exerted by transcription factor proteins that bind regulatory sequences of genes and affect their expression^1^. Elucidation of transcriptional regulatory systems is important for improving medicine and agriculture. For example, many diseases are associated with mutations in transcriptional regulators or in transcription factor binding sequences^1^. Changes in plant transcriptional regulation led to many modern crops and enabled large yield increases^2^. A better understanding of transcriptional regulation could help improve many agronomical traits such as biomass and resilience against pathogens^3^. Computational reverse engineering of gene regulatory networks has gained much attention over the last decade, driven by the emergence of large-scale gene expression analyses^4^,^5^. However, gene regulatory network inference remains a challenging task. This is in part due to the large amount of experimental noise and the large number of genes relative to the small sets of conditions in gene expression analyses^6^,^7^. In eukaryotes, gene expression levels are further affected by chromatin remodeling, and post-transcriptional and post-translational processes^8^. All these additional layers of regulation make inference of causal dependencies between genes from gene expression datasets alone even more difficult. While inference methods for *in silico* and prokaryotic datasets perform well^5^, inferring gene regulatory networks from eukaryotic datasets is more difficult^5^,^9^,^10^. As a consequence, heterogeneous data integration methods^11^ have emerged to construct more reliable eukaryotic biological networks for gene function prediction^11^,^12^,^13^ and gene regulatory network inference^14^.

Experimental validation could provide larger and more accurate gold standard data, which, in turn, could serve as training sets to improve prediction algorithms^10^,^15^. This makes experimentation an essential part of gene regulatory network discovery. Therefore, improving prediction accuracy to facilitate candidate selection for experimentation is one of the main challenges in gene regulatory network inference. This is particularly true for working with plants and animals that have a high number of potential regulatory interactions.

Here we present a semi-supervised network inference algorithm called GRACE (Gene Regulatory network inference ACcuracy Enhancement) to infer high-confidence gene regulatory networks. Our approach is based on Markov Random Fields^16^ and motivated by applications in Computer Vision such as image denoising or segmentation tasks^17^,^18^. Borrowing concepts from the field of Computer Vision to infer gene regulatory networks in prokaryotes has recently gained some attention^19^. GRACE enhances gene regulatory networks by integrating two complementary network data: (i) DNA binding based regulatory networks and (ii) co-functional networks. GRACE can evaluate the biological relevance of an inferred network and can be learned on sparse regulatory gold standards. We show GRACE’s potential to provide high confidence gene regulatory network predictions, compared to state of the art approaches, for *D. melanogaster* and *A. thaliana*. GRACE is freely available as R code, tutorial scripts as well as complementary datasets to generate the prioritized link predictions for *D. melanogaster* and *A. thaliana* at: https://github.com/mbanf/GRACE.

## Results

### An overview of the GRACE algorithm for gene regulatory network accuracy enhancement

**Figure 1:**
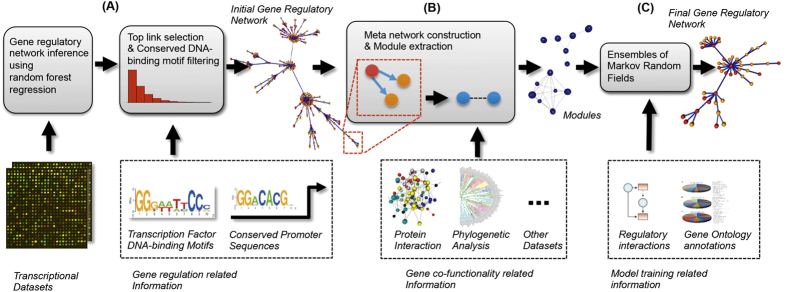
Overview of the GRACE algorithm: (**A**) GRACE integrates data relevant to gene expression and regulation to construct an initial gene regulatory network with transcription factors (red) and target genes (orange) represented as nodes and regulatory links as edges (blue). (**B**) Co-regulation related datasets are used to construct a meta network with regulatory links now represented as nodes (blue) to extract individual modules (tightly connected regulatory links). (**C**) An ensemble of Markov Random Fields is trained to re-evaluate and select regulatory links (blue) that form the final high confidence gene regulatory network.

Our algorithm, as illustrated in Fig. 1, first builds an initial gene regulatory network based on the integration of multiple heterogeneous, transcriptional-regulation related datasets (Fig. 1(A)). Data integration has proven necessary in the context of network inference for higher organisms^11^,^14^. A general challenge of data integration is the limited availability of different datasets. For example, transcription factor binding information can be powerful for establishing directed regulatory networks. However, binding information is available only for a limited number of transcription factors for most organisms^10^. Therefore, in order to produce an initial network, GRACE uses genome-wide datasets first, followed by a network refinement based on additional, more sparsely available, datasets. First, GRACE constructs an expression based gene regulatory network. To this end, GRACE implements a random forest regression model similar to the one used by GENIE3^20^, which is a state of the art gene expression based network inference algorithm. However, GRACE’s highly scalable random forest regression model performs several times faster than the one used in GENIE3 (see supplement for details and speed comparison). Subsequently, an empirical cumulative distribution over all link predictions is constructed and only the top 5% of all expression based link predictions are kept. Finally, these top predictions are further filtered with available transcription factor binding within conserved non-coding promoter sequences in order to obtain a direct binding based gene regulatory network (see methods).

GRACE then integrates co-function network data by constructing a *meta gene regulatory network* structure such that nodes in this meta network represent the regulatory links of the original network. Pairs of nodes in the meta network are connected if they contain a pair of target genes that share a common regulator in the original regulatory network (Fig. 1(B)). As a consequence, the meta network decomposes into individual modules that represent a group of genes that are co-regulated by an individual transcription factor. For each module, a connectivity measure between node pairs is established, which is based on the degree of co-functionality between their corresponding co-regulated gene pairs. To define this connectivity measure, GRACE expects, as second input, a co-function gene network, which can be a simple co-expression or protein interaction network or a more complex co-functional network constructed based on integration of multiple heterogeneous datasets^12^,^13^.

Based on the rationale that a regulator participating in a highly weighted regulatory interaction to one gene is more likely to regulate a second, closely co-functional gene, we model each module as a Markov Random Field, typically used in Computer Vision for image denoising or segmentation tasks^17^,^18^, to obtain a probability whether a node within a module should be kept or be removed (Fig. 1(C)). This corresponds to whether a link from the original network should be removed based on whether it facilitates a strong co-regulatory relationship. As a consequence, the original gene regulatory network is pruned. GRACE learns the hyperparameters of the Markov Random Field model from given gold-standard datasets in a semi-supervised fashion.

For model training, we propose a variation of the traditional *f*_1_-score as optimization criterion to simultaneously evaluate recovery rates of known regulatory links and biological relevance of the prioritized regulatory links (co-functional evidence based on Gene Ontology). This optimization criterion makes GRACE particularly suited for model learning in organisms for which on only a limited amount of regulatory gold standard data exists.

### Using GRACE to infer high-confidence developmental gene regulatory networks in *D. melanogaster* and *A. thaliana*

We used GRACE to generate gene regulatory networks for *D. melanogaster* and *A. thaliana* development (see methods). For *A. thaliana* we generated an initial gene regulatory network composed of 325 regulators, 4305 targets, and 10098 regulatory links (methods). For *D. melanogaster* we generated an initial gene regulatory network composed of 133 regulators, 8413 targets, and 17772 regulatory links (see methods).

To enhance the accuracy of the initial networks, we used the latest release of AraNet^12^ and FlyNet^13^, two genome-scale association networks constructed based on diverse data types, as the co-functional networks. As gold standards for model training and evaluation, for *A. thaliana*, we used the ATRM (Arabidopsis Transcriptional Regulatory Map)^21^ dataset (regulatory evidence) and an experimental Gene Ontology benchmark as provided by AraNet^13^ (co-functional evidence). For model evaluation we performed 100 rounds of hold out validation, based on *N* = 100 individually trained GRACE models, using 0.632% of the gold standard (regulatory and co-functional evidence) as training and the remaining 0.328% as non-overlap test set. Furthermore, we used two additional independent (i.e. not used during training) validation datasets, SUBA3 (co-localization evidence)^22^ and ARACYC (co-occurrence in metabolic pathways)^23^. For *D. melanogaster*, we used the REDfly dataset (regulatory evidence)^14^ and an experimental Gene Ontology benchmark as provided by FlyNet^12^ (co-functional evidence) for training and testing, as well as two independent validation datasets, ChIP binding (regulatory evidence) and Hi-C (chromatin contact conformation), both provided by ref. 14.

To evaluate the performance of GRACE’s accuracy enhancement process, we compared the average enrichment of gold standard recovery rates (for each test and independent validation dataset) in GRACE’s final prediction to the initial network across all hold out runs. In order to avoid putative performance biases caused by different network sizes, we selected the same number (as GRACE’s final prediction) from the top network links of the initial network across all 100 tests. For *A. thaliana*, GRACE’s propagation step increased prediction accuracy by 40% for co-functional gene pairs (p-value = 0.096, Fisher’s exact test), 125% for co-localized gene pairs (p-value < 1.1e–139, Fisher’s exact test), and 450% for genes in the same metabolic pathway (p-value < 0.0006, Fisher’s exact test). For *D. melanogaster*, GRACE’s propagation step increased prediction accuracy by 20% for co-functional gene pairs (p-value < 7.2e–5, Fisher’s exact test), 60% for Chip binding(p-value < 9.8e–7, Fisher’s exact test), and 20% for chromatin contact conformation (p-value < 0.01, Fisher’s exact test).

### Comparison of GRACE’s prediction accuracy to other algorithms

We compared GRACE’s predictions to two of the top performing gene regulatory network inference algorithms from a recent large-scale comparative analysis^5^: (*i*) the CLR (Context likelihood of relatedness) algorithm^24^, which is a mutual information based approach, corrects predictions based on the specific background distribution of all mutual information scores; and (*ii*) the tree-based regression method, GENIE3^20^. In addition to mutual information and regression based approaches, we tested GRACE against a partial correlation based method, called GGM (graphical gaussian model)^25^. In addition, we compared GRACE to two recently proposed algorithms that have been designed to incorporate additional data types beyond gene expression data: (*i*) the wGLASSO (weighted graphical Lasso)^26^ and (*ii*) iRafNet^27^. wGLASSO is an extension of the graphical Lasso. The graphical Lasso approach estimates the sparse inverse covariance matrix in a Gaussian graphical model by a lasso (L1) penalty. This matrix is then used to identify connections between pairs of genes. wGLASSO, instead of allowing only a single global penalty, allows for individual penalty values per gene pair based on additional evidence. iRafNet can be seen as an extension of the GENIE3 approach, as it adjusts the gene expression-based random forest regression so that putative regulators that are supported by additional information will be more frequently sampled during decision tree construction. We ran both wGLASSO and iRafNet using the conserved transcription factor binding information as well as AraNet^12^ (for *A. thaliana*) and FlyNet^13^ (for *D. melanogaster*) as additional network information. For wGLASSO, a penalty parameter *ρ* = 0.45 was selected based on simulations described in ref. 26. For iRafNet, default parameters were selected.

**Figure 2:**
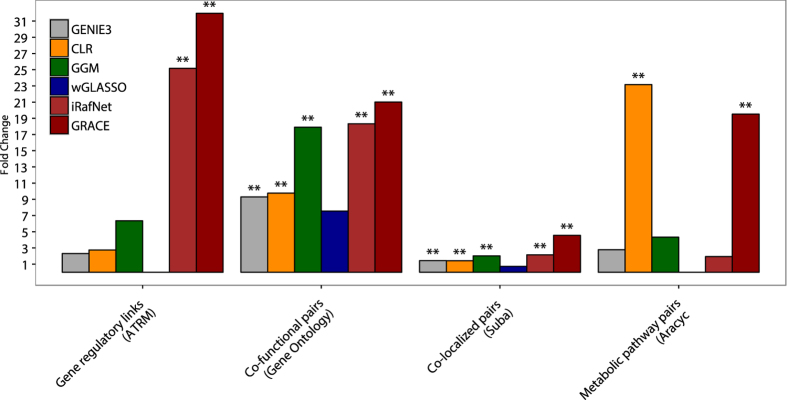
Each algorithm’s fold change enrichment of gold standard recovery rates over the two test (ATRM, Gene Ontology) and two independent validation (Suba, Aracyc) datasets for *A. thaliana* (p-value < 0.01 (**)).

**Figure 3:**
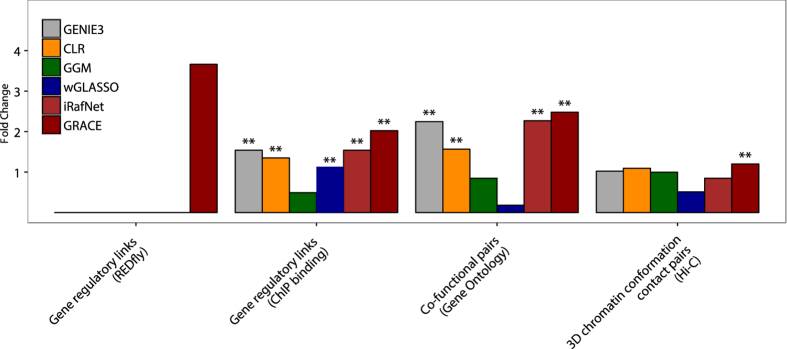
Each algorithm’s fold change enrichment of gold standard recovery rates over the two test (REDfly, Gene Ontology) and two independent validation (ChiP binding, Hi-C) datasets for *D. melanogaster* (p-value < 0.01 (**)).

We computed the enrichment of gold standard recovery rates on all datasets averaged across test and independent validation datasets of all hold out runs. For a fair comparison of regulatory link recovery rates, we selected the same number of links as predicted by GRACE from each compared method. For the co-regulated gene pair recovery analysis, we selected each algorithm’s number of co-regulated target gene pairs formed by these top links. To make performance assessment of each method comparable to each other, we used the maximum number of possible regulatory links and co-regulated gene pairs as background. These numbers correspond to the number of links within the fully connected regulatory as well as pairwise gene networks, defined by the number of regulators and genes per species. Based on these analyses we observe GRACE to produce more accurate predictions with respect to 4 out of 4 test datasets as well as 3 out of 4 independent validation datasets compared to all other methods in *A. thalana* (Fig. 2) as well as *D. melanogaster* (Fig. 3).

To evaluate the statistical significance of GRACE’s improvements, we compared GRACE’s performance to each individual method’s prediction across all test and independent validation datasets using Fisher’s exact test. Fold changes were computed between gold standard recovery rates of GRACE’s and all other methods, using same number of links as predicted by GRACE per each hold out test. GRACE’s predictions were more accurate with statistical significance (p-value < 0.05) in 9 out of 20 cases for *A. thaliana* (Table 1) and 8 out of 16 cases for *D. melanogaster* (Table 2). In addition, the methods compared had zero gold standard recovery rates in 8 cases (indicated by ‘—’).

Finally, we constructed an ensemble model for *D. melanogaster* and for *A. thaliana* combining link predictions of all 100 individual models for analysis. To build the ensemble model, each link *l* in the original gene regulatory network was kept, if its likelihood *L*(*x*_*l*_) > 0.5, i.e. if it has been predicted by more than 50 individual models. The *A. thaliana* network retained 7.8% (792) of the 10098 links from the initial network. For *D. melanogaster*, GRACE retained 40.3% (7164) of the links from the initial network predictions (Supplementary datasets). A topological analysis of the resulting network predictions by GRACE revealed several regulatory hubs. Furthermore, the distributions over the out-degree per regulator, representing its number of targets, followed a power law for both networks (data not shown), as expected for biological networks^28^. We evaluated the final ensemble model on the independent validation datasets for both species and compared their performance to five other methods. GRACE outperformed other methods with statistical significance in 8 out of 9 cases for *A. thaliana* (Table 3), and in 7 out of 9 cases for *D. melanogaster* (Table 4).

### GRACE recovers and predicts cell cycle control mechanisms during *A. thaliana* developmental programs

To further verify GRACE’s link predictions, we examined a subset of the inferred network related to *A. thaliana* cell cycle progression, a major driving force for plant growth. Given the essential role of the molecular machinery behind cell proliferation, there is a high degree of conservation among organisms^29^. This makes cell cycle control during various stages of plant development an ideal model system to evaluate the inferred regulatory network.

**Figure 4:**
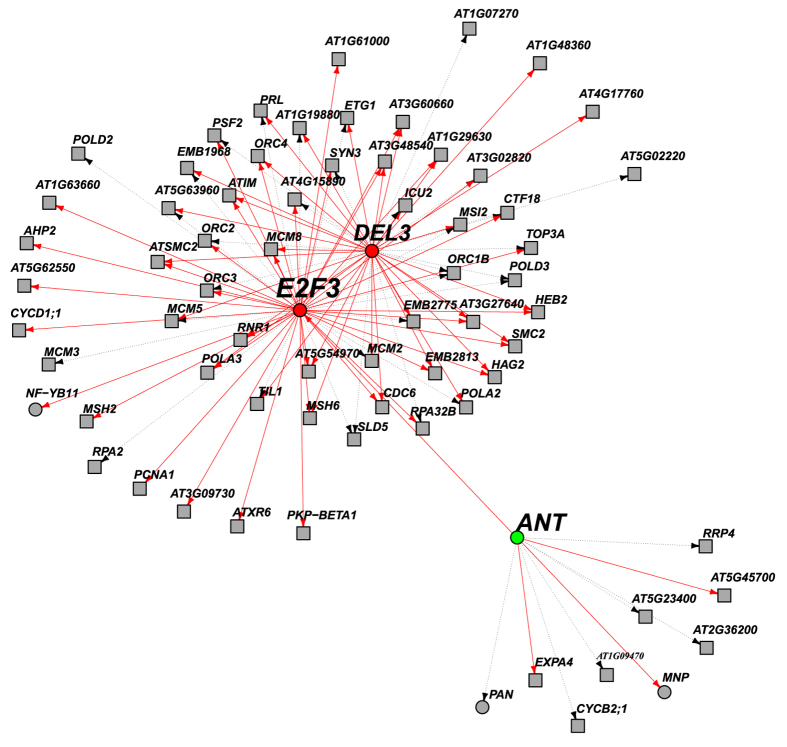
An auxin-controlled regulatory hub involved in cell cycle progression, including the E2F3/DEL3 complex (red) and ANT (green). Transcription factors and other genes are represented as circles and squares, respectively. Red solid lines between transcription factors and putative targets (transcription factors or other genes) represent regulatory links inferred only by GRACE, while black dashed lines denote regulatory links found both in the top 792 initial predictions and GRACE.

**Figure 5:**
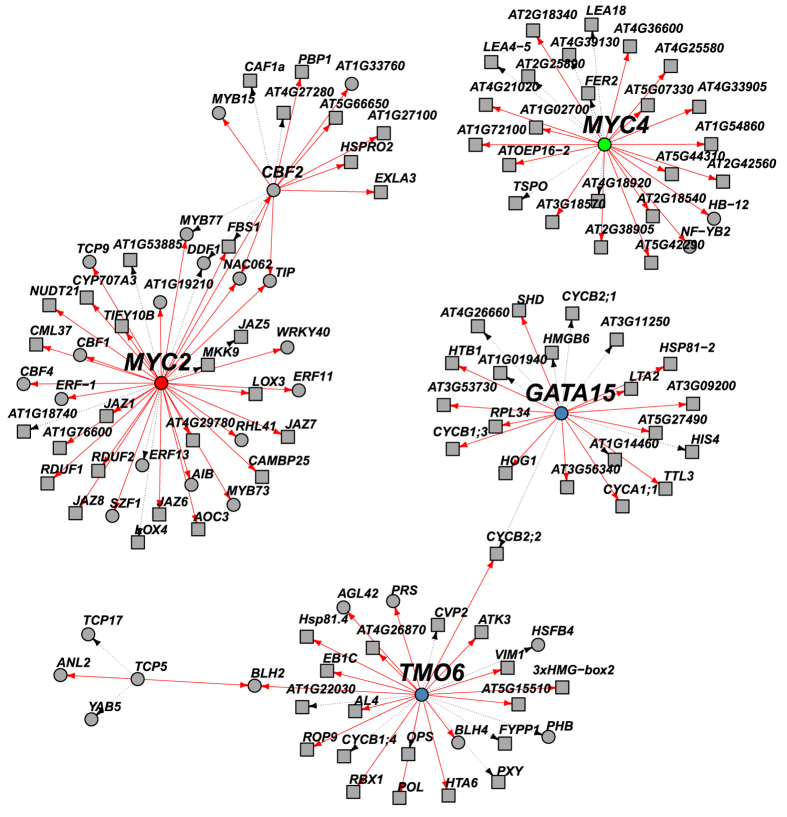
Jasmonates control leaf growth by repressing cell proliferation through MYC2/4 (red/green), cell division through GATA15 (blue) and vascular structure formation through TMO6 (blue). Transcription factors and other genes are represented as circles and squares, respectively. Red solid lines between transcription factors and putative targets (transcription factors or other genes) represent regulatory links inferred only by GRACE, while black dashed lines denote regulatory links found both in the top 792 initial predictions and GRACE.

GRACE recovered known control mechanisms. Several hormones play essential roles as signals for cell division, expansion and differentiation^30^. GRACE recovered well-characterized hub regulators such as E2F3, DEL3 (Fig. 4 (red)), and MYC2 (Fig. 5 (red)), known to be controlled by hormones such as auxin or jasmonic acid^31^. In particular, E2F3 represents a regulator for auxin-dependent cell cycle activation^32^. GRACE recovered many cell cycle related targets of E2F3, including those involved in chromosomal replication (e.g. ORC1-4, MCM2-5, Pola2-3, RNR1)^33^, DNA repair (e.g. AHP2, PCNA1, EMB1968, EMB2813, EMB2775, HEB2)^33^,^34^ and cell division (e.g. CYCD1;1, CDC6)^33^,^34^. In addition, two other known targets of E2F3 were recovered, ETG1 (E2F target gene 1), a conserved replisome factor that binds with MCM (Mini-Chromosome Maintenance complex) and is crucial for DNA replication^35^, as well as CTF18 (Chromosome Transmission Fidelity 18)^36^ that acts in synergy with ETG1 to establish sister chromatid cohesion during DNA replication^35^.

A second group of hormones influencing cell proliferation are jasmonates. For instance, during leaf development, jasmonates control leaf growth by repressing cell proliferation and the onset of endoreduplication. Within the signaling cascades that are triggered by jasmonates, JAZ (Jasomate-Zim Domain) repressor proteins play a central role, given their interaction with a broad array of transcription factors. GRACE identified two members of the basic helix-loop-helix transcription factor family, MYC2 (Fig. 5 (red)) and MYC4 (Fig. 5 (green)), both reported to be direct targets of JAZ proteins^31^, to act as regulatory hubs within the inferred network. MYC2 is known to form homo- or heterodimers with MYC3 and MYC4 and fine-tune the jasmonate signaling pathway by regulating not only the expression of transcriptional activators that function downstream from MYC2, but also the JAZ repressors that act upstream from MYC2^37^,^38^. GRACE recovered this negative feedback loop mechanism between MYC2 and multiple JAZ repressors (JAZ1, JAZ3, JAZ5, JAZ6, JAZ7 and JAZ8). In addition, GRACE recovered several other targets of MYC2, including CML37^39^ as well as LOX3 and LOX4 (Lipoxygenase 3 and 4)^40^, both encoding allene-oxide cyclases and involved in jasmonic acid biosynthesis.

In addition to recovering known relationships, GRACE made several novel predictions. Two examples are the predicted hub regulators, GATA15 and TMO6 (Fig. 5 (blue)). Members of the GATA transcription factor family influence many developmental processes downstream of several hormone signaling pathways, including auxin and gibberellin^41^. Here GRACE predicts multiple cell division related targets of GATA15 (e.g. CYCA1;1,CYCB1;3, CYCB2;1, CYCB2;2) as well as histone genes (e.g. HIS4, HTB1).

TMO6 (Target of Monopteros 6) is involved in vein formation and vascular development^42^. In general, the formation of vascular patterns involves specific regulation of a number of cellular processes, including cell proliferation^43^. In particular, modifications of cell proliferation patterns alter the number of higher order vein structures^43^. GRACE predicts regulatory relationships between TMO6 and several cell division (e.g. CYCB1;4, CYCB2;2) as well as histone (e.g. HTA6) and histone binding genes (e.g. AL4). In addition, GRACE predicts regulatory links among TMO6, VIM2 and PXY. VIM2 (Variant in Methylation 1) is crucial in maintaining chromatin structure during cell division^44^ and PXY encodes a receptor-like kinase that maintains cell polarity necessary for the orientation of cell division during plant vascular development^45^. Here the predicted regulatory link between TMO6 and PXY could explain a direct control mechanism of TMO6’s involvement in vascular structure formation.

A major auxin-controlled regulator during cell division is ANT (Aintegumenta) (Fig. 4 (green)). ANT controls plant organ cell number and organ size^46^. GRACE predicted regulatory links between ANT and cell division (CYCB2;1) and cell expansion (EXPA4) related genes, which have not been linked previously. In addition, GRACE predicted the bZIP transcription factor PAN (Perianthia) to be a direct target of ANT. This putative regulatory link is supported by experimental evidence in which reduced expression levels of PAN were reported in SEU/ANT double mutants^47^. PAN plays a central role in flowering and acts as a direct regulator of AG (Agamous)^48^. Given that a regulatory effect of ANT on AG has been reported previously^49^, we hypothesize that ANT and PAN, together with AG, form a feed forward loop mechanism, a common regulatory motif in biological networks^50^.

## Discussion

Here we introduce the GRACE algorithm, a learning-based approach to construct and enhance the accuracy of predicted gene regulatory networks, especially for eukaryotic organisms, based on the concept of heterogeneous data integration with ensembles of Markov Random Fields. GRACE’s design principle to consider co-regulation enables the integration of two complementary information, DNA binding based regulatory networks and co-functional networks.

We have shown GRACE’s potential to provide more accurate network predictions on eukaryotic datasets compared to state of the art methods, including those that have also been designed to integrate additional information into the inference process.

GRACE’s major advantage is its additional degree of freedom. While traditional integrative models are controlled by only a single global parameter *γ*, i.e. the regulatory link strength threshold, GRACE can use co-functionality to propagate likelihoods of genes being co-regulated by the same set of regulators and re-evaluate and prioritize individual regulatory interactions. In contrast, in traditional models such as those based on logistic regression^14^, co-functionality information is typically used after model training for validation purposes. Here GRACE uses a second parameter *λ* to control the influence of this additional biological prior information to make local adjustments of the regulatory network, while simultaneously guaranteeing *λ* to be consistent on a global scale. This also considers the fact that co-functionality datasets are typically only available for a subset of the genome. In addition, model training based on regulatory and co-functional evidence recovery simultaneously allows for the application of supervised and semi-supervised inference schemes even on sparse datasets.

GRACE complements traditional clustering approaches for regulatory network inference^51^,^52^ that cluster highly co-expressed target genes to identify regulators of gene clusters. GRACE provides several advantages that circumvent some of the limitations of these approaches^7^. For example, given the sparsity of experimental DNA binding information (e.g. those derived from Chip-seq experiments), clustering approaches predict motifs de novo per gene cluster, based on the assumption of the entire cluster being co-regulated by a set of transcription factors. This assumption can lead to a high rate of false positive predictions, even when additional co-functional cues are used to define gene clusters.

In summary, GRACE’s approach to re-evaluate the links of an initial network based on pairwise link strength propagation across a co-functional network is beneficial in several respects: (i) it considers the partial nature of co-functional datasets; (ii) it is less prone to over-predictions due to large clusters of highly co-expressed genes; and (iii) it allows for the inference of non-linear, e.g. antagonistic, co-regulatory relationships. GRACE’s performance evaluation scheme will help distinguish multiple methods, which perform similarly on the regulatory evidence alone, and assist in the selection of more biologically relevant candidates for experimentation.

## Methods

### Integrative gene regulatory network inference

To construct the initial *A. thaliana* developmental gene regulatory network, we integrated three types of datasets. First, we incorporated conserved non-coding sequences within 2000 bp promoter regions of 17610 *A. thaliana* genes^53^. Conserved non-coding promoter sequences were shown to be reliable predictors of regulatory elements controlling gene expression^14^,^53^. Second, we added DNA binding predictions within these sequences for 120 transcription factors as provided by Van de Velde *et al*.^53^. In addition, we predicted binding within these promoter sequences for curated experimental DNA binding motifs of an additional set of 270 transcription factors^54^,^55^,^56^. Therefore, we mapped the curated binding elements to all conserved non-coding promoter elements within the 17610 *A. thaliana* genes using the bioconductor TFBSTools package (p-value threshold *p* < 0.001). As a result, we obtained a regulatory blueprint of 390 regulators and 17610 targets with 219000 link predictions. Third, we added an expression atlas of *A. thaliana* development^57^ comprising RNA samples from 83 tissues and developmental stages. The expression data was used to derive a condition specific co-expression network. The expression dataset had already been normalized using Robust Multichip Averaging (RMA)^57^. Subsequently, we averaged tissue and developmental stage specific experimental replicates. Finally, a variance based filtering (using the genefilter R package) was applied to remove genes that exhibited little variation across all tissues and developmental stages.

To infer a gene regulatory network from the gene expression dataset, we implemented a highly scalable and robust tree based regression (see Supplement for details), which decomposes network inference into a separate regression problem for each possible target gene. It uses tree-based regression to calculate an importance measure for each predictor, which is used as an indicator for a link to be present between the regulator and the target gene. Given the large number of regulators in *A. thaliana*, we ran our tree based regression models with 5000 decision trees for each target gene. This is to ensure that all regulators are selected multiple times during random forest specific bootstrap aggregation in order to provide stable predictions per target gene. We predicted expression-based regulatory links for all transcription factors, not just those with available DNA binding information. This avoids biasing predictions towards regulators with binding information, which might not be the main regulators for a given gene expression dataset. Subsequently, an empirical cumulative distribution function was constructed over all resulting regulatory predictions. In general, only a small subset of all possible predicted regulatory links are expected to be true interactions, based on the general assumption of gene regulatory networks to be sparse^5^,^58^. Therefore, users can select a minimum threshold, with respect to the empirical cumulative distribution, to extract a sub-network. For all our evaluations we retained all predictions beyond the 95^*th*^ percentile of the distribution, obtaining a total of 263000 regulatory link predictions for *A. thaliana*. Combining the DNA binding and the gene expression based gene regulatory networks we obtained an initial gene regulatory network composed of 325 regulators, 4305 targets, and 10098 regulatory links.

For *D. melanogaster*, we obtained physical DNA binding based regulatory interactions (420787 links) based on experimentally defined DNA binding occupancy profiles from ChIP studies for 76 transcription factors, as well as conserved DNA binding motifs for 139 transcription factors^14^. In addition, gene expression profiles across the developmental time-course, also provided by^14^, were used as input to our tree based regression model to infer a gene expression based network, which was subsequently filtered based on the 95^*th*^ percentile threshold, obtaining a total of 69081 regulatory link predictions. Combining the DNA binding and the gene expression based gene regulatory networks we obtained an integrative network of 133 regulators and 8413 targets with 17772 regulatory links. In the integrative gene regulatory network a *regulatory link l* is defined as a regulatory interaction between a regulator *r* (e.g. a transcription factor) and a target gene *g*, i.e. *l*:*r* → *g*. The strength of this interaction is denoted as *υ*_*grnl*_.

### Constructing a meta gene regulatory network

Given the set of regulatory links *l*, we formulate the concept of a *meta gene regulatory network* that describes connections between pairs of links, i.e. *l* ↔ *l*′. A connection between two links *l, l′* is defined based on the co-regulation principle, i.e. two different target genes *g, g*′ are controlled by the same regulator *r*, i.e. *l* ↔ *l*′ = *r* → {*g, g*′}. The weight of such a connection 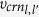 is based on a distance metric that combines two measures, which are assumed to reflect co-regulation, i.e.:


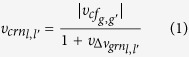


with


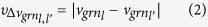


where 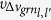 denotes the absolute pairwise distance between the weights 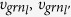 of connected regulatory links *l* and *l*′. As 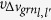 increases, 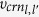, and in turn the likelihood of pairwise co-regulation, decreases. 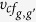 denotes the level of co-functionality of the gene pair *g, g*′, as given by the gene functional network. In the simplest case, this could be the Pearson’s correlation coefficient measured over the expression levels of the gene pair *g, g*^′^.

Given this meta gene regulatory network, we now extract modules of connected regulatory links, as illustrated in Fig. 1. Each module *u* represents a group of target genes *g* that are pair-wise co-regulated by a specific transcription factor *r*. Subsequently, per module, we employ a variation of Prim’s algorithm^59^, called *maximum spanning tree* algorithm. For a weighted undirected graph, which each module represents, the algorithm constructs a tree-structured graphical model, including every node but using only a minimal subset of the highly weighted connections with respect to 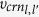 between nodes. The approach removes loops from the module graph, which is beneficial for modeling influences between links.

Individual links *l* that are not connected to any other link are also retained and represented as individual (single link) modules.

### Modeling co-regulation using Markov Random Fields

To model co-regulatory effects within each module *u* we employ a *Markov Random Field* approach. A Markov Random Field is represented as an undirected graph *G* = (*V, E*), which implements a local independence assumption referred to as Markov property. The Markov property imposes a node to be independent of any other nodes given all its direct neighbors, i.e.:


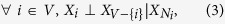


Here, *N*_*i*_, {*j*|{*i, j*} ∈ *E*} denotes the set of immediate neighbors of node *V*_*i*_ in the graph *G*. Eq. 3 postulates that *X*_*i*_ and *X*_*V*−{*i*}_ are independent given 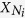. An important notion in the model is that of a *clique c*. It is defined as a fully connected subset of nodes within the graph, which is considered maximal if it is not contained within any other larger clique^16^. The corresponding joint probability distribution satisfies Eq. 3, i.e. the local Markov property. According to Hammersley-Clifford theorem^16^, it can be factorized as:


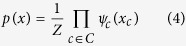


where *Z* denotes a normalizing factor, often referred to as the *partition function*, since the potential functions *ψ*_*c*_(*x*_*c*_) do not have to represent probabilities, as opposed to Bayesian networks. By considering only potential functions that satisfy *ψ*_*c*_(*x*_*c*_) ≥ 0, one can ensure that *p*(*x*) ≥ 0. A common model for Computer Vision applications is the (pairwise) Markov Random Field that factorizes into unary *ψ*_*l*_(.) as well as pairwise clique potentials 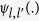 to incorporate correlation effects between neighboring pixels:


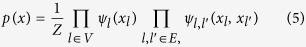


The Markov Random Field model defines a probability distribution over the output variables *x* via an energy function *E*(*x*). It is convenient to express *ψ*_*l*_(*x*_*l*_) and 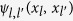 as exponentials, i.e.:


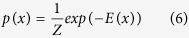


with


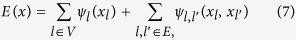


Given the normalizing factor *Z* we can model any type of (non-probabilistic) energy functions *E*(*x*). Therefore, we now define the energy function used in our model to represent the effect of co-regulation in gene regulatory network inference, i.e.:


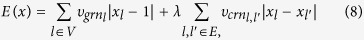


Here, *υ*_*grnl*_ denotes the weight of each regulatory link *l* as provided by the individual gene regulatory network inference method used. 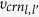 is the connectivity strength between two regulatory links *l, l*^′^ as defined in Eq. 1. *x*_*l*_ and 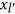 represent the state variables *x* ∈ {0, 1}, i.e., whether a regulatory link *l* will be in the final network (*x*_*l*_ = 1) or whether it will be excluded (*x*_*l*_ = 0). Therefore, our model favors links *l* with high weights, i.e. *υ*_*grnl*_, as well as strongly connected pairs of regulatory links to be in the same state. *λ* represents a global penalty, besides the pairwise penalty term 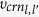. It follows that single link modules are represented without the pairwise clique potential 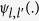. In order to solve the above energy function *E*(*x*)_*u*_ for each module *u*, we face two major connected challenges: First, since the less-confident links will predominate confident links, we might run the risk of removing a highly confident link based on a larger number of connected less-confident links. In conventional machine learning terms, this problem can be seen as a classification task with highly imbalanced datasets. Second, it is generally not known in advance what weight *υ*_*grnl*_ should represent a suitable value for uncertainty in classifying *l* as either *x* = 1 (valid regulatory link) or *x* = 0 (invalid). Since such a value cannot be extracted from the original inferred regulatory network itself, we address these challenges by formulating a learning problem in order to predict a suitable model hyperparameter, *γ* > 0, so that if *υ*_*grnl*_ ≤ *γ, l* would be more likely to be removed unless it is connected to a highly confident regulatory link *l*′. Accordingly, we extend Eq. 8 by *γ*, i.e.:


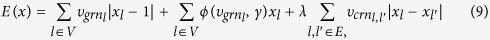


with


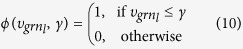


For single link modules, Eq. 9 reduces to 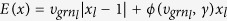. As *γ* also depends on *λ*, which penalizes the influence of connected links, we can define a hyperparameter set *θ* that is to be optimized for a given gold standard, i.e.:


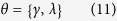


In our graphical model, we condition on links *l* with *υ*_*grnl*_ > *γ*, given that undirected graphical models are closed under conditioning^60^. This ensures that *l* can be recovered if connected to a highly confident regulatory link *l*′ but will not be removed if connected to a multitude of low confidence links.

### An f-score based optimization criterion to integrate regulatory evidence and biological relevance

The quality of an inferred network is typically evaluated by interpreting the problem as a binary classification task and predicting edges as either present or absent. In the context of gene regulation, gold standards typically only contain positive class labels, i.e. examples of interacting gene pairs. To generate a set of negative examples, we followed the standard approach that interprets the absence of a link in this matrix as a non-interacting gene pair. We used precision vs. recall curves to measure an algorithm’s performance^61^. We used this measure over the receiver operator measure because the latter has been shown to paint an overly optimistic view of an algorithm’s performance if there is a large skew in the class distribution^61^. This is the case for the task of gene regulatory network inference in which the number of putative true negatives far outnumbers the most likely true positives.

A major challenge in gene regulatory inference lies in the sparsity of suitable regulatory link gold standard data for model training or evaluation, as only a small fraction of curated gold standard datasets is typically recovered over hundreds or even thousands of predicted high confidence links. This is particularly true for integrative inference with partial DNA binding information, when a given gold standard is first filtered to exclude links based on regulators without available DNA binding motifs. This sparsity makes the selection of a suitable subset of high-confidence predictions difficult, as well as the comparison of different inference methods on these small high confidence subsets. Since it is generally assumed that genes in a gene regulatory network are preferentially linked to genes involved in similar biological processes, Gene Ontology annotations have been used to validate network predictions^14^. Therefore, we propose a combined *f*_*β*_-score based optimization to select a high-confidence network for maximizing the recovery of known regulatory links and biological relevance during model learning:


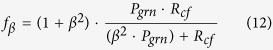


Here precision *P* still denotes the number of gold standard regulatory links over the number of predictions but recall *R* is now defined as the number of co-regulated gene pairs with similar experimental co-function annotation (Gene Ontology) over the total number of co-regulated gene pairs with similar experimental co-function annotation. For robustness, we follow the definition of co-regulation as proposed in ref. 14, i.e. gene pairs regulated by shared regulators (defined by a Jaccard’s coefficient higher than 50%). Here, Jaccard’s coefficient computes the intersection (shared) over the union of involved regulators per gene pair.

Given that precision and recall describe different biological evidences and with a maximum recall of 1, we also scale precision, which sets its maximum value to 1. This is to compensate for the sparsity of the recovered regulatory information in gold standard data (as discussed in the main test) in order to define reasonable minimum network sizes. Adjustments in *β* then allow for a user to shift emphasis from precision (based on regulatory evidence) to recall (based on co-functional evidence). During our evaluations, we chose the *f*_1_-score for both *A. thaliana* and *D. melanogaster* for optimization.

### Deriving regulatory link probabilities using ensembles of Markov Random Fields

Learning *θ* represents a typical case of hyperparameter tuning, which refers to learning algorithm-specific model parameters. Hyperparameter tuning applies to not only supervised methods but also algorithms such as clustering, which are typically considered to be unsupervised. To learn suitable hyperparameters *θ* while optimizing for *f*_*β*_, we implemented a coarse-to-fine optimization strategy. First, we use *grid search*^62^ to reduce the range of parameters to a smaller sub-space. Subsequently, we employ *Simulated Annealing*, an optimization strategy known for its ability to avoid local optima^63^, to identify the exact model parameters within this smaller parameter sub-space. We select the ranges of parameter sets *θ* for hyperparameter tuning as *γ* ∈ [*υ*_*grn*0%_,*υ*_*grn*100%_] with *υ*_*grn*100%_ denoting the predicted interaction strength level at the 100% mark of all edges considered in the meta gene regulatory network. The ranges of *λ* can be defined by the user to enforce an upper and lower limit on the influence of the co-functional network on the re-evaluation of the regulatory link predictions. In our experiments we set *λ* ∈ [0.001, 2.5]. Given that multiple equivalent, similar plausible local optima might exist, we apply a conservative optimization scheme to select the local optima around the lowest value for *λ*. This imposes an additional upper constraint on *λ*, preventing the system from over-predicting master regulators. We observe convergence of our trained models well within the given hyperparameter ranges.

GRACE evaluates a Markov Random Field per module *u* for a given *θ*, whose results, when combined with probabilities of individual Markov Random Fields for every other module, describe the likelihood *L*(*x, θ*) over the entire meta gene regulatory network for all links *l*, i.e.:


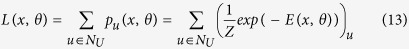


Here, 
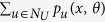
 combines the non-overlapping results per each module *u. θ* is the same for all modules *u*. In order to solve the above energy function *E*(*x*)_*u*_ for each Markov Random Field, as defined in Eq. 13, for each module *u* for a specific parameter set *θ*, we employ *Belief Propagation*^64^, also called *sum-product* algorithm, to obtain the marginal probabilities per node (a link *l* in the original network) within each *u*. After convergence, a regulatory link *l* in the original gene regulatory network is kept within the filtered network, only if its marginal probability *p*_*l*_ equals *p*_*l*_ > 0.5.

For model evaluation, we perform *N* rounds of hold-out validation. Therefore, we train *N* = 100 individual GRACE models, per model sampling 0.632% of the modules *u* and the corresponding gold standard data, using the remaining 0.328% as test set to compute the average gold standard recovery enrichment. For the final model, to increase robustness of our predictions and avoid over-fitting, we use a bootstrapping aggregation-based ensemble strategy, as typically used within the random forest framework^65^. Therefore, an unweighted ensemble model is constructed by averaging over all *N* models. This ensemble model then defines a final likelihood per link *l*:


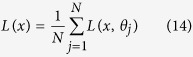


Based on this ensemble model, a link *l* is selected if *L*(*x*_*l*_) > *ε*_*p*_. In our experiments we use *ε*_*p*_ = 0.5.

## Additional Information

**How to cite this article:** Banf, M. and Rhee, S. Y. Enhancing gene regulatory network inference through data integration with markov random fields. *Sci. Rep.* **7**, 41174; doi: 10.1038/srep41174 (2017).

**Publisher's note:** Springer Nature remains neutral with regard to jurisdictional claims in published maps and institutional affiliations.

## Supplementary Material

Supplemental Methods

Supplementary Dataset 1

Supplementary Dataset 2

## Figures and Tables

**Table 1:** Comparative analysis of GRACE’s predictions versus all other methods for each test (ATRM, Gene Ontology) and independent validation (Suba, Aracyc) dataset for *A. thaliana*.

Methods/Datasets	ATRM	Gene Ontology	Suba	Aracyc
GRACE vs GENIE3	FC = 7.7 (p = 0.058)	FC = 2.3 (p = 0.0002)	FC = 3.3 (p = 1.3e–154)	FC = 7.4 (p = 0.0001)
GRACE vs CLR	FC = 7.1 (p = 0.07)	FC = 2.2 (p = 0.04)	FC = 3.3 (p = 3.7e–134)	FC = 0.8 (p = 0.48)
GRACE vs GGM	FC = 3.6 (p = 0.14)	FC = 1.2 (p = 0.47)	FC = 2.2 (p = 2e–41)	FC = 2.5 (p = 0.11)
GRACE vs wGLASSO	—	FC = 3.2 (p = 0.39)	FC = 7.7 (p = 1.93e–17)	—
GRACE vs iRafNet	FC = 1.3 (p = 0.72)	FC = 1.2 (p = 0.42)	FC = 2.1 (p = 3.3e–76)	FC = 5.3 (p = 0.015)

Fold changes (FC) and p-values based on Fisher’s exact test. ‘−’ indicates zero gold standard recovery rates of the compared method.

**Table 2:** Fold change comparative analysis of GRACE predictions versus all other methods for each test (REDfly, Gene Ontology) and independent validation (ChiP binding, Hi-C) dataset for *D. melanogaster*.

Methods/Datasets	REDFLY	ChiP Binding	Gene Ontology	Hi-C
GRACE vs GENIE3	—	FC = 1.3 (p = 9.3e–7)	FC = 1.1 (p = 0.19)	FC = 1.2 (p = 0.09)
GRACE vs CLR	—	FC = 1.5 (p = 1.7e–12)	FC = 1.6 (p = 0.0001)	FC = 1.1 (p = 0.39)
GRACE vs GGM	—	FC = 4.1 (p = 4.2e–77)	FC = 2.9 (p = 2.7e–10)	FC = 1.2 (p = 0.13)
GRACE vs wGLASSO	—	FC = 1.5 (p = 6.7e–12)	FC = 9.7 (p = 0)	FC = 2.4 (p = 0.16)
GRACE vs iRafNet	—	FC = 1.3 (p = 9.5e–7)	FC = 1.1 (p = 0.24)	FC = 1.4 (p = 0.0008)

Fold changes (FC) and p-values based on Fisher’s exact test. ‘—’ indicates zero gold standard recovery rates of the compared method.

**Table 3:** Comparative analysis of GRACE’s predictions (ensemble model) vs all other methods for each independent validation (Suba, Aracyc) dataset for *A. thaliana*.

Methods/Datasets	Suba	Aracyc
GRACE vs GENIE3	FC = 3.7 (p = 5.4e–322)	FC = 9.8 (p = 6.3e–06)
GRACE vs CLR	FC = 3.3 (p = 2.746077e–187)	FC = 0.9 (p = 0.5)
GRACE vs GGM	FC = 2.3 (p = 2.9e–82)	FC = 4.0 (p = 0.03)
GRACE vs wGLASSO	FC = 7.7 (p = 1.9e–17)	—
GRACE vs iRafNet	FC = 2.2 (p = 8.0e–152)	FC = 5.2 (p = 0.015)

Fold changes (FC) and p-values based on Fisher’s exact test. ‘—’ indicates zero gold standard recovery rates of the compared method.

**Table 4:** Comparative analysis of GRACE’s predictions (ensemble model) versus all other methods for each independent validation (ChiP binding, Hi-C) dataset for *D. melanogaster*.

Methods/Datasets	ChiP Binding	Hi-C
GRACE vs GENIE3	FC = 1.3 (p = 3.3e–07)	FC = 1.1 (p = 0.16)
GRACE vs CLR	FC = 1.5 (p = 3.5e–13)	FC = 1.1 (p = 0.5)
GRACE vs GGM	FC = 4.1 (p = 7.9e–86)	FC = 1.3 (p = 0.05)
GRACE vs wGLASSO	FC = 1.5 (p = 6.5e–12)	FC = 2.4 (p = 0.16)
GRACE vs iRafNet	FC = 1.3 (p = 1.7e–07)	FC = 1.4 (p = 0.0009)

Fold changes (FC) and p-values based on Fisher’s exact test.
