# Supplementary material for: Enhancing gene regulatory network inference through data integration with markov random fields
Source: Sci Rep. 2017 Feb 1;7:41174. doi: 10.1038/srep41174 (PMC5286517; doi:10.1038/srep41174)
Supplement: Supplemental Methods [file srep41174-s1.pdf]

Supplemental methods

Enhancing gene regulatory network inference through data integration with markov random fields

Michael Banf*, and Seung Y. Rhee*

Department of Plant Biology, Carnegie Institution for Science, 93405 Stanford, USA
*Corresponding authors: mbanf.research@gmail.com / srhee@carnegiescience.edu

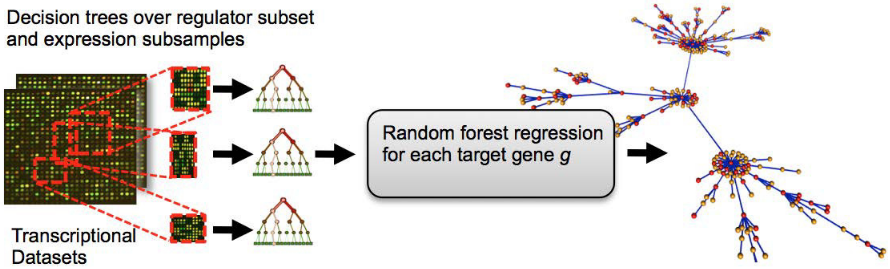

Figure 1. Gene regulatory network inference from gene expression data based on random forest regression.

Regression-based approaches decompose the network inference task into separate regression problems for each gene in the network in which the expression values of a particular target gene are predicted using all other genes as possible predictors ([1]), as illustrated in Fig. 1. This formulation is equivalent to the more general problem of feature selection in machine learning ([2]). Tree-based regression approaches, such as random forests ([3]), can handle complex interaction structures as well as highly correlated input variables and provide an inherent measure of variable importance. They rank among the best performing ensemble-based machine learning approaches for classification or regression tasks ([2]). Huynh-Thu et al. ([1]) introduced GENIE3, a random forest based gene regulatory network inference algorithm. It was one of the top performers in the DREAM5 network inference challenge ([4]).

Applying the concept of random forest regression to gene regulatory network inference, D represents a gene expression dataset with the input variables N_r being all putative regulator genes r (e.g. transcription factors). N_s denotes the set of gene expression values (e.g. RNA samples from different conditions). For each gene g, a number N_{tree} of decision trees is grown over different subsets of N_s (Fig. 1). *decrease of Gini impurity* (*DGI*) is used as a criterion for node splitting ([3]) and selecting the splitting predictor, i.e. a putative regulator gene r_i. Within each tree, the Gini information gain (IG) of r_i at node n, $IG(r_i,n)$, is the difference between the impurity at the node n and the weighted average of impurities at each child node of n, i.e., $IG(r_i,n) = DGI(r_i,n) - w_L IG(r_i,n_L) - w_R IG(r_i,n_R)$, with n_L and n_R being the left and right child nodes of n. w_L and w_R are the ratios of the number of instances at the left and right child nodes to the number of instances at node n. At each node, a random subset k_r of regulators is evaluated for node splitting based on $IG(r,n)$. Subsequently, predictions of all individual subset based decision trees are aggregated to rank all putative regulatory links $r \rightarrow g$.

To implement this concept, the R implementation of the state of the art algorithm, GENIE3 ([1]), at its core, employs the `randomforest` library ([5]) for random forest based classification and regression. In contrast, GRACE is designed to use the recently proposed `ranger` library ([6]) that addresses two bottlenecks in the decision tree regression procedure, *i* the considerations of node splitting candidates given all input variables, in our case the number of putative regulators N_r and *ii* drawing the k_r candidate splitting features per node from N_r. Both aspects become crucial in gene regulatory network inference, when the number of regulators N_r increases, as is the case for species with larger genomes. To speed up the regression procedure `ranger` harnesses two different splitting algorithms to sort the feature values beforehand and accesses them by their index, and to retrieve and sort values of the input variables while splitting. It exploits Knuth's algorithm for sampling without replacement[7] to optimize the selection of the k_r candidate splitting features per node.

Table 1 shows a side by side speed and accuracy comparison between GENIE3's and GRACE's random forest regression procedures. For a fair comparison, we implemented a parallelized version of the original GENIE3 algorithm. In this parallel version, we harness the fact that inference can be decomposed as individual regression problem per target, and use the `foreach` package to parallelize GENIE3's core for loop over all target genes. Gene expression data and experimentally validated gold standard data were collected from the DREAM5 challenge ([4]) for an *in silico* benchmark. The number of regulators r, target genes g, and sample sizes s for this dataset were r:195,s:805,g:1643. In addition, we used *A. thaliana* to evaluate speed differences on a species with a higher number of regulators. Therefore we used an expression atlas of *A. thaliana* development[8] and the ATRM (Arabidopsis Transcriptional Regulatory Map)[9] as experimental gold standard. The number of regulators r, target genes g, and sample sizes s for this dataset were r:1439/s:1388/g:22591). Table 1 shows accuracies based on Area under Receiver Operator curve (AUROC) as well as Area under Precision Recall curve (AUPR) for $k_r = \sqrt{N_r}$ and $N_{tree} = 1000$, as suggested by ([1]). For both datasets, we observe identical accuracy for GENIE3's and GRACE's random forest regression, while GRACE's being up to 11.5 times faster.

Method	GENIE3's tree regression AUPR/AUROC/TIME	GRACE's tree regression AUPR/AUROC/TIME	GRACE's tree regression Speedup
in silico	0.2911 / 0.833 / 4.7h	0.2923 / 0.833 / 0.8h	5.5 x
A. thaliana	0.0520 / 0.692 / 555.2h	0.0521 / 0.693 / 48.4h	11.5 x

Table 1. AUPR / AUROC scores as well as computation times of random forest regression frameworks used within GENIE3 and GRACE

References

1. V. A. Huynh-Thu, et al., Inferring regulatory networks from expression data using tree-based methods, PLoS One 5 (9). doi:10.1371/journal.pone.0012776.
2. Y. Saeys, et al., A review of feature selection techniques in bioinformatics, Bioinformatics 23 (19) (2007) 2507–17. doi:10.1093/bioinformatics/btm344.
3. L. Breiman, Random forests, Mach. Learn. 45 (1) (2001) 5–32. doi:10.1023/A:1010933404324.
4. D. Marbach, et al., Wisdom of crowds for robust gene network inference, Nat Methods 9 (8) (2012) 796–804. doi:10.1038/nmeth.2016.
5. A. Liaw, M. Wiener, Classication and regression by randomforest, R News 2 (3) (2002) 18–22.
6. M. N. Wright, A. Ziegler, ranger: A fast implementation of random forests for high dimensional data in c++ and r, Journal of Statistical Software.
7. D. Knuth, The Art of Computer Programming, Vol. 2, Addison-Wesley, 1985.
8. M. Schmid, et al., A gene expression map of arabidopsis thaliana development, Nat Genet 37 (5) (2005) 501–6. doi:10.1038/ng1543.
9. J. Jin, et al., An arabidopsis transcriptional regulatory map reveals distinct functional and evolutionary features of novel transcription factors, Mol Biol Evol 32 (7) (2015) 1767–73. doi:10.1093/molbev/msv058.
